# Anomalous Sub‐Band Gap Emission at Room Temperature in Mixed‐Dimensional WS_2_/Cu_2_O Heterostructures

**DOI:** 10.1002/smll.75662

**Published:** 2026-09-21

**Authors:** Osamah Kharsah, Jonah von Kuczkowski, Ahmed Shahin, Rouaa Albalkhi, Aya Al‐Amwi, Ibrahim Titi, Yossarian Liebsch, Oliver Altenhoff, Ulrich Hagemann, Anke Hierzenberger, Kevin Musselman, Marika Schleberger

**Affiliations:** ^1^ Faculty of Physics and CENIDE University of Duisburg‐Essen Duisburg Germany; ^2^ Mechanical Engineering University of Waterloo Waterloo Canada; ^3^ Interdisciplinary Center for the Analytics on the Nanoscale (ICAN) and CENIDE University of Duisburg‐Essen Duisburg Germany

**Keywords:** 2D materials, interfacial defects, metal oxides, ${\mathrm{ws}}_{2}$

## Abstract

Practical implementation of two‐dimensional (2D) transition‐metal dichalcogenides (TMDCs) in novel optoelectronics requires a fundamental understanding of carrier dynamics and interfacial states at 2D/3D heterostructures. Herein, a mixed‐dimensional p‐n junction based on monolayer tungsten disulfide (${\mathrm{WS}}_{2}$) and cuprous oxide (${\mathrm{Cu}}_{2}O$) is investigated. An anomalous emission (AE) at 1.66 eV is identified at room temperature, which is in the same spectral range as oxygen‐related ${\mathrm{Cu}}_{2}O$ defect luminescence, but is enhanced at the heterointerface under 457 nm excitation. While this sub‐bandgap feature resembles the spectral signature of an interlayer exciton (ILX), its combined excitation‐wavelength, power, temperature, and bias dependence suggests that the emission is predominantly defect‐mediated and modified by the ${\mathrm{WS}}_{2}$/${\mathrm{Cu}}_{2}O$ interface. External modulation of the AE intensity is demonstrated via vertical bias, yielding an approximately 45% maximum‐to‐minimum modulation of the normalized AE/${\mathrm{Cu}}_{2}O$ PL area ratio over the applied bias range from −2 V to +2 V at 93 K. This modulation is asymmetric, showing a correlation between AE quenching and photocurrent generation under reverse bias. The spectral response peaks near the open‐circuit condition, marking a regime of efficient carrier accumulation and interfacial trapping. These findings identify ${\mathrm{WS}}_{2}$/${\mathrm{Cu}}_{2}O$ as a model hybrid interface for probing electrically tunable defect‐mediated recombination in mixed‐dimensional heterostructures.

## Graphical abstract

Monolayer WS2 on Cu2O produces a room‐temperature sub‐bandgap emission from the hybrid interface. Spectroscopy and electrical bias measurements indicate that this emission arises primarily from WS2‐modified Cu2O defect states rather than interlayer excitons. The emission can be modulated by vertical voltage, revealing competition between defect recombination and photocurrent extraction.

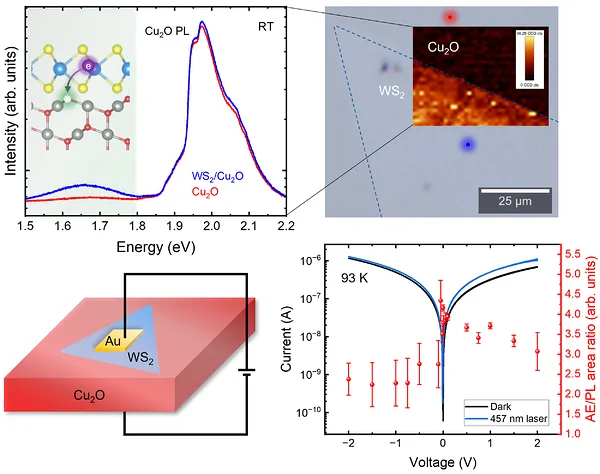

## 1 Introduction

The integration of two‐dimensional (2D) transition‐metal dichalcogenides (TMDCs) with semiconducting metal oxides offers a versatile platform for next‐generation optoelectronics, such as solar cells [1], light‐emitting diodes [2, 3], and photodetectors [4], combining the light‐matter interaction of TMDC monolayers with the carrier densities of bulk metal oxides [5, 6, 7]. Among these, cuprous oxide (${\mathrm{Cu}}_{2}O$) is of particular interest due to its inherent p‐type conductivity, high absorption coefficient, and the unique physics of its parity‐forbidden bandgap [8, 9, 10]. While recent studies have successfully integrated ${\mathrm{Cu}}_{2}O$ with TMDCs such as ${\mathrm{MoS}}_{2}$ and ${\mathrm{WSe}}_{2}$ to realize high‐responsivity phototransistors and photocatalytic systems [4, 11, 12], the synergy between ${\mathrm{Cu}}_{2}O$ and monolayer tungsten disulfide (${\mathrm{WS}}_{2}$), an optically active 2D TMDC with a direct bandgap of approximately 2.4 eV [13], remains unexplored.

As a mixed‐dimensional 2D/3D p–n heterostructure, the ${\mathrm{WS}}_{2}/{\mathrm{Cu}}_{2}O$ interface is expected to be governed by complex charge‐transfer processes that dictate both optical response and electrical transport. In such systems, interfacial states and defects often act as critical intermediaries. However, while localized states in bare ${\mathrm{Cu}}_{2}O$ are well‐studied [14, 15, 16, 17], their role in charge trapping and defect emission within a hybrid 2D/3D environment has not been explored. Understanding these interactions is essential for tailoring the radiative pathways in hybrid metal oxide devices [18, 19]. In particular, a hybrid 2D/3D interface offers the prospect of optically addressing and electrically tuning the occupation of these states, making oxide‐surface transients accessible to optical and electrical probes in hybrid optoelectronic structures [20, 21].

In this work, we report the discovery of an anomalous emission (AE) at 1.66 eV at room temperature in mixed‐dimensional p‐n heterostructures composed of a CVD‐grown ${\mathrm{WS}}_{2}$ monolayer and commercially available single‐crystalline (111) ${\mathrm{Cu}}_{2}O$. This feature is enhanced in the ${\mathrm{WS}}_{2}$‐covered ${\mathrm{Cu}}_{2}O$ region relative to adjacent bare ${\mathrm{Cu}}_{2}O$ and resides relatively deep within the sub‐bandgap region of both materials. Given the predicted staggered type‐II band alignment of this system, the discovery of the AE poses a fundamental question: is this signal a localized defect‐mediated emission specifically enhanced by the presence of the ${\mathrm{WS}}_{2}$ monolayer, or does it represent the formation of a mixed‐dimensional interlayer exciton (ILX)?

To unravel the physical origin of this anomalous emission in this heterostructure, we performed a systematic spectroscopic investigation of its excitation dynamics and electric‐field response at room temperature and 77 K. Here, we report that the AE in ${\mathrm{WS}}_{2}$/${\mathrm{Cu}}_{2}O$ exhibits a sub‐linear power dependence (k≈0.3) and high sensitivity to excitation energy, suggesting a mechanism driven by hot‐electron populations. These dynamics are consistent with a carrier‐localization regime at the interface. By contrasting the emission's resilience under vertical bias with the expected behavior of a Coulomb‐bound ILX at 77 K, we provide further evidence for interfacial defect emission.

Ultimately, these findings suggest that the sub‐bandgap emission is dominated by an interfacial defect‐mediated recombination pathway rather than by an ILX. While 2D/3D heterostructures are often studied from the perspective of how the substrate modifies the 2D semiconductor, the present work emphasizes the inverse role: monolayer ${\mathrm{WS}}_{2}$ acts as an atomically thin sensitizing layer that grants access to ${\mathrm{Cu}}_{2}O$ interfacial states. Rather than competing with ${\mathrm{Cu}}_{2}O$/TMDC photodetectors in overall macroscopic responsivity, this study broadens the role of the heterostructure beyond a conventional device architecture into a highly sensitive, spectrally resolved probe of oxide surface‐state dynamics.

## 2 Results

### 2.1 Compositional and Optical Characterization

We performed fundamental compositional and optical characterization to analyze the chemical and phononic properties of the ${\mathrm{WS}}_{2}$/${\mathrm{Cu}}_{2}O$ heterostructure. X‐ray photoelectron spectroscopy (XPS) measurements were performed to confirm the composition of the ${\mathrm{Cu}}_{2}O$ and ${\mathrm{WS}}_{2}$/${\mathrm{Cu}}_{2}O$. Our results show the Cu 2p core level (Figure S1a,b), the O 1s core level (Figure S1d,e), and W 4f (Figure S1g,h) for ${\mathrm{Cu}}_{2}O$ and ${\mathrm{WS}}_{2}$/${\mathrm{Cu}}_{2}O$ in good agreement with the literature [10, 22, 23]. Consistent with studies on ${\mathrm{Cu}}_{2}O$ (111) surface defects [24], we observe an increase in the oxygen lattice state ($O_{\mathrm{lat}}$) upon heterojunction formation [22]. While bare oxide exhibits a dominant oxygen‐related defect signature ($O_{d}$) at 531.9 eV [25, 26], accounting for ≈81% (in some interpretations vacancy‐caused OH groups [27]), this ratio decreases in ${\mathrm{WS}}_{2}$‐covered ${\mathrm{Cu}}_{2}O$ to ≈70%. The decrease in the $O_{d}$ component suggests that the ${\mathrm{WS}}_{2}$ monolayer passivates or electronically modifies coordinatively unsaturated oxygen sites or adsorbed OH groups [24, 27].

Furthermore, the S 2p core levels (Figure S1c,f,i) reveal a complex sulfur environment. In the ${\mathrm{WS}}_{2}$/${\mathrm{Cu}}_{2}O$ heterostructure, alongside the characteristic ${\mathrm{WS}}_{2}$ sulfide peaks (S 2$p_{3/2}$ and S 2$p_{1/2}$ at 162.6 and 163.8 eV, respectively), we observe additional states at 168.5 eV and 169.7 eV. These higher binding energy features are indicative of sulfate (${\mathrm{SO}}_{4}^{2-}$) species [28]. Crucially, the XPS measurement of a separate ${\mathrm{Cu}}_{2}O$ control crystal that had not been subjected to transfer (Figure S1c) shows no detectable sulfur signal, whereas a bare ${\mathrm{Cu}}_{2}O$ region measured after the ambient ${\mathrm{WS}}_{2}$ growth and transfer process (Figure S1f) exhibits distinct sulfate‐related features. Because the control measurement was performed on a different crystal, we cannot fully exclude that the ${\mathrm{Cu}}_{2}O$ substrate used for the heterostructure already contained trace sulfur before transfer. Nevertheless, the comparison makes the introduction of sulfur‐containing species during the ambient growth and transfer process plausible. Previous studies have shown that sulfate species can form when trace sulfur residues interact with ${\mathrm{Cu}}_{2}O$ surfaces under ambient conditions [29, 30]. Importantly, the oxygen‐related defect luminescence in the 1.6–1.7 eV range is also observed in the pre‐transfer ${\mathrm{Cu}}_{2}O$ control crystal, for which no sulfur signal is detected by XPS. Sulfate species are therefore not required for the underlying sub‐bandgap emission. The present data do not, however, exclude a secondary influence of transfer‐induced sulfur‐containing species on the intensity or spectral characteristics of the interface‐modified emission.

Next, we used Raman spectroscopy to study the phononic properties of ${\mathrm{Cu}}_{2}O$ and the ${\mathrm{WS}}_{2}$/${\mathrm{Cu}}_{2}O$ heterostructure. Figure 1a shows the Raman modes for ${\mathrm{Cu}}_{2}O$ (red) and ${\mathrm{WS}}_{2}$/${\mathrm{Cu}}_{2}O$ (blue). For ${\mathrm{Cu}}_{2}O$, the positions of the Raman modes are close to experimental literature values [31, 32], with the main 2$E_{u}$ (silent) mode and $T_{1u}$ (IR active) mode being at 219 and 149 ${\mathrm{cm}}^{-1}$, respectively. Additionally, the $T_{2g}$ mode related to the motion of oxygen ions is measured at 496 ${\mathrm{cm}}^{-1}$, while the $T_{1u}$ mode that describes the relative Cu–O lattice motion (Cu–O stretching and O–Cu–O bending) is found at 646 ${\mathrm{cm}}^{-1}$ [33].

**FIGURE 1:**
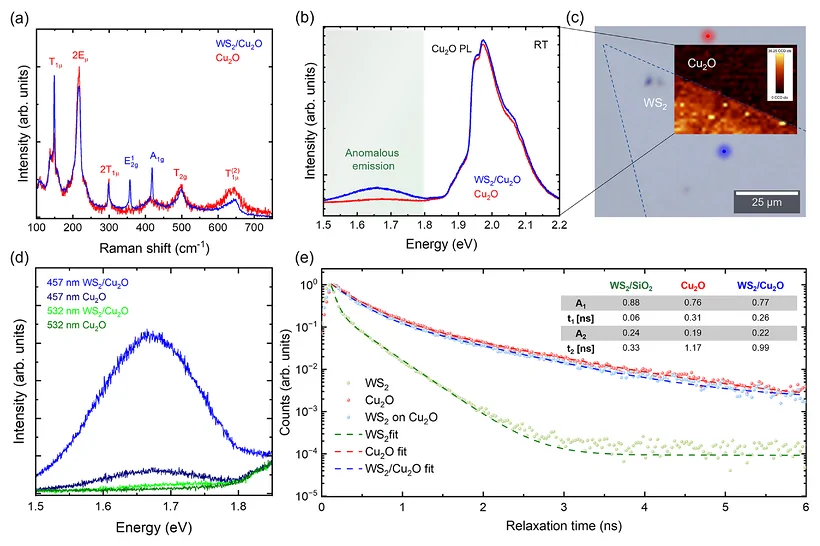
(a) Raman modes shown for bare ${\mathrm{Cu}}_{2}O$ (red) and the ${\mathrm{WS}}_{2}$/${\mathrm{Cu}}_{2}O$ heterostructure (blue). The measurement was conducted using a 457 nm laser. (b) Logarithmically plotted room‐temperature PL spectra are shown for both bare ${\mathrm{Cu}}_{2}O$ (red) and the ${\mathrm{WS}}_{2}$/${\mathrm{Cu}}_{2}O$ heterostructure (blue), measured with a 457 nm laser. We observe the AE for ${\mathrm{WS}}_{2}$/${\mathrm{Cu}}_{2}O$ at approximately 1.66 eV. (c) Spatially resolved PL mapping confirms that the 1.66 eV emission is enhanced in regions where the ${\mathrm{WS}}_{2}$ monolayer covers ${\mathrm{Cu}}_{2}O$. While bare ${\mathrm{Cu}}_{2}O$ exhibits similar PL characteristics, the intensity of the emission around 1.66 eV is primarily found to be higher in ${\mathrm{WS}}_{2}$/${\mathrm{Cu}}_{2}O$. Red and blue dots indicate the positions of the measured PL spectra seen in (b). (d) Room‐temperature PL spectra of the AE region measured using 532 nm (green) and 457 nm (blue) excitation wavelengths for ${\mathrm{WS}}_{2}$/${\mathrm{Cu}}_{2}O$ and ${\mathrm{Cu}}_{2}O$. (e) Exciton dynamics of ${\mathrm{WS}}_{2}$ (green), ${\mathrm{Cu}}_{2}O$ (red), and ${\mathrm{WS}}_{2}$/${\mathrm{Cu}}_{2}O$ heterostructure (blue) with the lifetimes and their amplitudes fitted using a bi‐exponential decay function.

The transfer of ${\mathrm{WS}}_{2}$ onto ${\mathrm{Cu}}_{2}O$ adds the Raman signature of ${\mathrm{WS}}_{2}$ to the Raman spectrum, with the out‐of‐plane $A_{1g}$ mode being at 417.9 ±0.3 ${\mathrm{cm}}^{-1}$ and the in‐plane $E_{2g}^{1}$ mode being at 356.9 ±0.5 ${\mathrm{cm}}^{-1}$. While the $A_{1g}$ peak experiences an average upshift of only +1.4 ${\mathrm{cm}}^{-1}$ compared to ${\mathrm{WS}}_{2}$ on the ${\mathrm{SiO}}_{2}$ growth substrate (Figure S2b), the $E_{2g}^{1}$ mode exhibits an average upshift of +1.7 ${\mathrm{cm}}^{-1}$. Given the lattice constants of ${\mathrm{WS}}_{2}$ (a=3.16 Å) and ${\mathrm{Cu}}_{2}O$ (111) (a=3.02 Åfor the Cu‐sublattice) [34, 35], this shift is consistent with compressive strain induced by the substrate lattice structure.

Furthermore, we will outline some of the most important optical properties of ${\mathrm{Cu}}_{2}O$ before the discussion of the ${\mathrm{WS}}_{2}$/${\mathrm{Cu}}_{2}O$ heterostructure. This will subsequently allow a deeper understanding of the anomalous emission. The PL spectrum of ${\mathrm{Cu}}_{2}O$, shown in Figure 1b (red), is dominated by a strong emission centered around 2.0 eV. This corresponds to the direct transitions from the valence band to the conduction band at the Γ‐point of the Brillouin zone [35]. Due to parity conservation in ${\mathrm{Cu}}_{2}O$, the direct dipole transition is forbidden. Therefore, this direct gap recombination PL is usually explained by dipole‐allowed phonon‐assisted transitions at room temperature. This leads to a PL emission that is highly susceptible to temperature changes. Due to strong spin‐orbit coupling in ${\mathrm{Cu}}_{2}O$, the valence band is split into two bands ($\Gamma_{7^{+}}$ and $\Gamma_{8^{+}}$) with an energetic separation of ≈0.13 eV [35, 36], which contributes to the rise of the observed PL peak.

Nevertheless, a faint defect emission is visible around 1.69 eV in the PL spectrum at room temperature, as seen in Figure 1b (red) and better visualized in Figure 1d (457 nm ${\mathrm{Cu}}_{2}O$). This defect emission is usually attributed to oxygen‐related defects in ${\mathrm{Cu}}_{2}O$, such as vacancies or interstitials [37, 38, 39].

At room temperature, we observe this peak in the ${\mathrm{Cu}}_{2}O$ spectrum when excited at 457 nm (Figure 1d), whereas it disappears when excited at 532 nm. Thus, to populate the oxygen‐related defect states in sufficient numbers, a higher excitation energy is required, allowing the detection of the DE at room temperature. This could be explained by the 457 nm wavelength exciting hot electrons that relax to these defect states, while most 532 nm photoexcited electrons relax to the band edge and contribute to the dipole‐allowed phonon‐assisted transitions. In addition, the 457 nm laser could allow for resonant injection directly into the defects, as reported by Gloystein et al. [15].

Furthermore, the higher excitation energy could allow electrons to be excited from the lower $\Gamma_{8}^{+}$ valence band and ultimately excite a higher number of hot electrons. Based on this, we establish that the increased transition probability achieved at higher laser energies is essential for observing optical sub‐bandgap processes in ${\mathrm{Cu}}_{2}O$.

### 2.2 Anomalous Emission at Room Temperature

Next, we turn our attention to the ${\mathrm{WS}}_{2}$/${\mathrm{Cu}}_{2}O$ heterostructure, where CVD‐grown ${\mathrm{WS}}_{2}$ monolayers are mechanically transferred onto ${\mathrm{Cu}}_{2}O$ (see Experimental Section 4). As seen in Figure 1b (blue), the usual ${\mathrm{Cu}}_{2}O$ emission dominates the PL spectrum, suggesting that the ${\mathrm{WS}}_{2}$ PL is quenched on the p‐type ${\mathrm{Cu}}_{2}O$ substrate. Significantly, we identify a distinct room‐temperature anomalous PL emission at approximately 1.66 eV, which lies below the bandgaps of both ${\mathrm{WS}}_{2}$ and ${\mathrm{Cu}}_{2}O$. This anomalous emission (AE) is found in the same energy range as the aforementioned ${\mathrm{Cu}}_{2}O$ DE. Yet, its higher‐intensity signal stems from the ${\mathrm{WS}}_{2}$ flake, as seen in spatially resolved PL mapping in Figure 1c. In comparison, observing spatially resolved PL of adjacent bare ${\mathrm{Cu}}_{2}O$ with focus on the usual ${\mathrm{Cu}}_{2}O$ PL emission shows an emission stemming from the entirety of the substrate (Figure S2d), thus differentiating the PL emission from the observed AE. Similar to the DE in bare ${\mathrm{Cu}}_{2}O$, this emission is only detectable at room temperature when the ${\mathrm{WS}}_{2}$/${\mathrm{Cu}}_{2}O$ material system is excited using the 457 nm laser, as seen in Figure 1d, suggesting that hot‐electron or high‐energy excitation processes may contribute to its formation. The comparison in Figure 1b,d was performed under identical optical conditions for ${\mathrm{WS}}_{2}$‐covered ${\mathrm{Cu}}_{2}O$ and bare ${\mathrm{Cu}}_{2}O$ regions. The spatially resolved PL map in Figure 1c further shows that the 1.66 eV intensity is spatially correlated with the ${\mathrm{WS}}_{2}$ monolayer.

To obtain a qualitative picture of possible charge‐transfer pathways, we combine literature values for electron affinity and bandgaps [40, 41] with Kelvin‐probe force microscopy (KPFM) data (Figure S3a,b). This analysis suggests a staggered, type‐II‐like alignment (Figure S3c). Because the KPFM measurements were performed under ambient conditions, the extracted work functions and band offsets should be regarded as approximate. Adsorbed water, hydroxyl groups, transfer residues, and local interface dipoles can influence the measured contact potential difference. Therefore, the KPFM‐derived alignment is used only to illustrate a plausible charge‐transfer scenario, not as a definitive determination of the buried ${\mathrm{WS}}_{2}$/${\mathrm{Cu}}_{2}O$ band offsets. Importantly, because these ambient conditions match those of our PL measurements, they provide the most relevant representation of the active heterostructure during optical operation.

Interestingly, our KPFM results reveal a higher work function for the n‐type ${\mathrm{WS}}_{2}$ monolayer compared to the p‐type ${\mathrm{Cu}}_{2}O$. This leads to a built‐in potential with an electric field pointing from ${\mathrm{Cu}}_{2}O$ to ${\mathrm{WS}}_{2}$, opposite to a conventional p‐n junction. We note that these values are estimates, and the actual experimental alignment likely differs due to system complexities. Specifically, hydroxyl groups and compressive strain in the ${\mathrm{WS}}_{2}$ layer (leading to band renormalization) are expected to modify the final ${\mathrm{WS}}_{2}$/${\mathrm{Cu}}_{2}O$ band offsets. Based on this alignment, two potential mechanisms for the AE formation arise: interlayer exciton (ILX) formation at the ${\mathrm{WS}}_{2}$/${\mathrm{Cu}}_{2}O$ interface, or interfacial defect emission enhanced by the ${\mathrm{WS}}_{2}$.

In the case of the ILX, electrons and holes populate the CB and VB of both ${\mathrm{WS}}_{2}$ and ${\mathrm{Cu}}_{2}O$ upon excitation. Due to the extreme quantum confinement and high density of photoexcited electrons in ${\mathrm{WS}}_{2}$, the type‐II alignment permits spatially indirect electron–hole pairs, potentially forming interlayer excitons between the CB of ${\mathrm{WS}}_{2}$ and the VBs of ${\mathrm{Cu}}_{2}O$. This could further be enhanced by the charge injection from ${\mathrm{WS}}_{2}$ to ${\mathrm{Cu}}_{2}O$ and vice versa due to the type‐II band alignment. While this scenario is feasible, it is important to keep in mind that to date, only a handful of 2D/3D heterostructures have been shown to host an ILX at room temperature, including bulk ${\mathrm{PbI}}_{2}$/bilayer ${\mathrm{WS}}_{2}$ and monolayer bismuth/SiC [18, 42, 43].

Alternatively, the AE could stem from electrons generated in the ${\mathrm{WS}}_{2}$ layer transitioning to interfacial defect states in the ${\mathrm{Cu}}_{2}O$ before radiatively decaying. Such a mechanism was reported for ${\mathrm{WS}}_{2}$/graphene interfaces [44]. In this case, the ${\mathrm{WS}}_{2}$ would act as an efficient light‐absorbing layer, where the neutral excitons are then dissociated due to the type‐II band alignment and the built‐in potential ($V_{\mathrm{bi}}$) at the interface.

To gain insight into the exciton dynamics of ${\mathrm{Cu}}_{2}O$ and the ${\mathrm{WS}}_{2}$/${\mathrm{Cu}}_{2}O$ heterostructure, we performed time‐resolved photoluminescence measurements. The data were fitted using $I\left(t\right)=y_{0}+A_{1}e^{-\frac{t-t_{0}}{t_{1}}}+A_{2}e^{-\frac{t-t_{0}}{t_{2}}}$. Figure 1e shows a slight decrease in the lifetimes of ${\mathrm{WS}}_{2}$/${\mathrm{Cu}}_{2}O$ (0.26 ns for $t_{1}$ and 0.99 ns for $t_{2}$) compared to ${\mathrm{Cu}}_{2}O$ (0.31 ns for $t_{1}$ and 1.17 ns for $t_{2}$). On its own, the ${\mathrm{WS}}_{2}$ monolayer exhibits much faster exciton dynamics near our detection limit (0.06 ns for $t_{1}$ and 0.33 ns for $t_{2}$), leading to a slightly faster exciton relaxation in the heterostructure compared to bulk ${\mathrm{Cu}}_{2}O$. Our results seen in Figure 1e are less consistent with long lifetimes commonly reported for ILX [45, 46], and more consistent with defect‐mediated recombinations that alter the exciton dynamics at the ${\mathrm{WS}}_{2}$/${\mathrm{Cu}}_{2}O$ interface [47]. The ${\mathrm{WS}}_{2}$ lifetime extracted on the reference substrate contains a component close to the instrumental response limit of the time‐correlated single photon counting (TCSPC) setup. Therefore, the absolute value of the fastest ${\mathrm{WS}}_{2}$ decay component should not be overinterpreted. The TRPL data are used only qualitatively to compare the overall decay dynamics of ${\mathrm{Cu}}_{2}O$ and ${\mathrm{WS}}_{2}$/${\mathrm{Cu}}_{2}O$ under identical measurement conditions.

In the following section, we expand on the characteristics found experimentally using low‐temperature and power‐dependent PL. The data gained from these experiments will help us better understand the mechanism of the AE.

### 2.3 The Origin of the Anomalous Emission

In this section, we present additional measurements that contribute to understanding the physical origin of the AE and to distinguishing between ILX formation and defect‐mediated recombination. We begin our discussion by introducing low‐temperature PL and power‐dependent PL to probe the excitonic behavior of the AE in ${\mathrm{WS}}_{2}$/${\mathrm{Cu}}_{2}O$ and the DE in ${\mathrm{Cu}}_{2}O$.

Starting with the low‐temperature PL response of ${\mathrm{WS}}_{2}$/${\mathrm{Cu}}_{2}O$ (Figure 2a), the AE shows a blue shift from 1.66 eV at 293 K to 1.7 eV at 77 K. Decreasing the temperature significantly increases AE intensity, while slightly decreasing the FWHM (Figure S4b). A weaker temperature‐dependent progression is measured for the DE in bare ${\mathrm{Cu}}_{2}O$ PL (Figure S4a), with a shift from around 1.69 eV at 293 K to 1.7 eV at 77 K. To quantify this enhancement, we compared the integrated 1.60–1.75 eV emission intensity from the heterostructure to adjacent bare ${\mathrm{Cu}}_{2}O$ regions. This analysis reveals that the sub‐bandgap emission is enhanced by a factor of 1.24 ± 0.62 (24% on average) at room temperature, and by a factor of 2.55 ± 0.68 (155% on average) at 77 K. The high uncertainty stemming from the statistical analysis could be attributed to non‐radiative recombination processes locally occurring in the CVD‐grown ${\mathrm{WS}}_{2}$ as established in the literature [13, 48].

**FIGURE 2:**
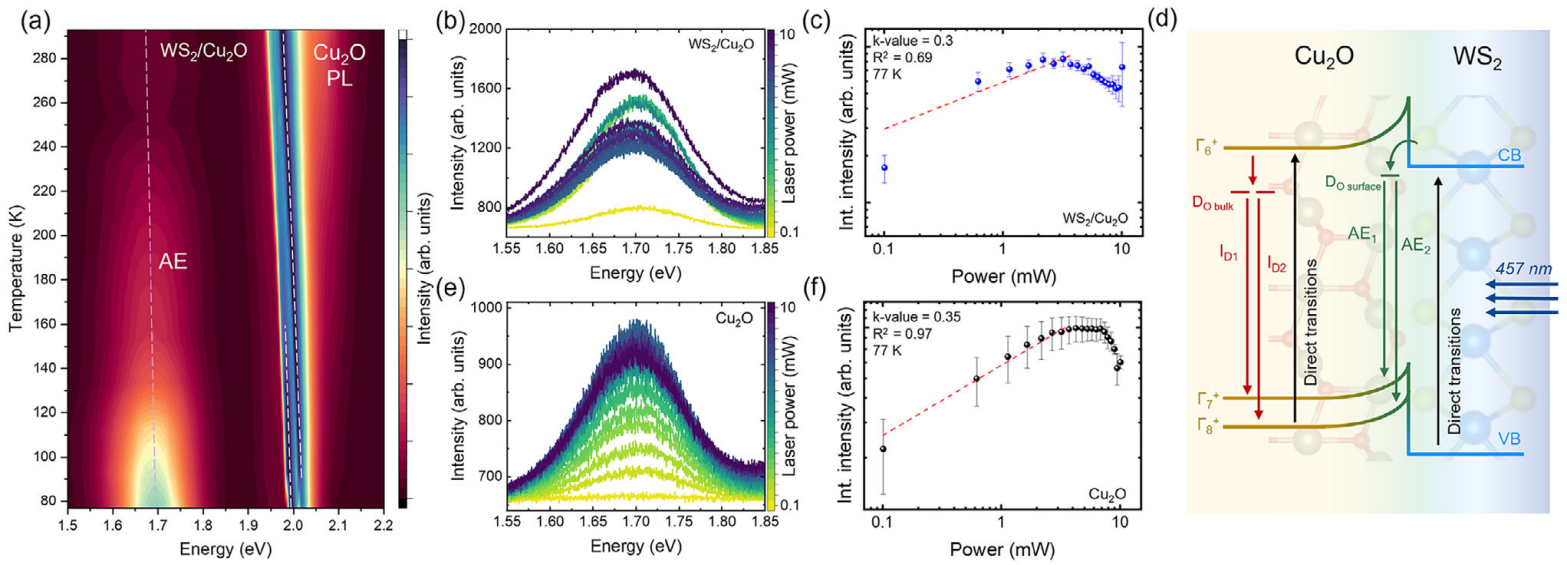
(a) Heatmap of the temperature‐dependent PL of ${\mathrm{WS}}_{2}$/${\mathrm{Cu}}_{2}O$. The colormap shows the progression of the excitons associated with ${\mathrm{Cu}}_{2}O$, as well as the combined contribution of the AE and DE. (b) The power‐dependent combined AE and DE of ${\mathrm{WS}}_{2}$/${\mathrm{Cu}}_{2}O$ with the power ranging from 0.1 to 10 mW, measured with 457 nm excitation at 77 K. (c) Power‐dependent PL analysis of the AE in the ${\mathrm{WS}}_{2}$/${\mathrm{Cu}}_{2}O$ heterostructure. (d) A schematic band diagram illustrating the optical transitions in the ${\mathrm{WS}}_{2}$/${\mathrm{Cu}}_{2}O$ system, such as the direct transitions in ${\mathrm{WS}}_{2}$ and ${\mathrm{Cu}}_{2}O$, as well as the charge transfer from ${\mathrm{WS}}_{2}$ to ${\mathrm{Cu}}_{2}O$ (green) across the interface, and the DE in ${\mathrm{Cu}}_{2}O$ (red). (e) Power‐dependent PL response of bare ${\mathrm{Cu}}_{2}O$, showing the DE around 1.7 eV. (f) Power‐dependent PL analysis of the DE in bare ${\mathrm{Cu}}_{2}O$. Fitting the power range yields effective exponents of k≈0.30 for the ${\mathrm{WS}}_{2}$/${\mathrm{Cu}}_{2}O$ AE and k≈0.35 for the bare ${\mathrm{Cu}}_{2}O$ DE. The comparable sublinear exponents indicate trap‐mediated recombination in both cases, while the faster saturation of the ${\mathrm{WS}}_{2}$/${\mathrm{Cu}}_{2}O$ emission is consistent with modified trap occupation at the heterointerface.

Comparing the temperature‐dependent intensities of the AE to the DE (Figure S4c) shows a stronger increase in AE intensity. However, the general trend for both emissions is that of a gradual increase in intensity until around 170 K, before a steeper increase commences. The FWHM shows some similarity in both emissions. While the DE starts with a higher FWHM, it decreases to reach levels similar to the AE by 170 K. These results are consistent with defect‐mediated recombination that is highly temperature‐dependent for the AE. Overall, the similar behavior for the AE and DE at 77 K suggests similar physical origins for both emissions.

Further information about the excitonic nature of AE and DE can be obtained using power‐dependent PL measurements, which are shown in Figure 2b,e for ${\mathrm{WS}}_{2}$/${\mathrm{Cu}}_{2}O$ and bare ${\mathrm{Cu}}_{2}O$, respectively. While the power‐dependent intensity progression of the ${\mathrm{Cu}}_{2}O$ DE is gradual, the intensity of ${\mathrm{WS}}_{2}$/${\mathrm{Cu}}_{2}O$ AE increases sharply at the beginning and fluctuates, as depicted in Figure 2c,f. This indicates a rapid intensity rise followed by early saturation in the AE. Conversely, the DE follows a gradual trend where new states are continuously excited, using higher laser power. To gain more insight into the recombination mechanism leading to this behavior, we use the following power law between the intensity (I) and excitation power (P):

(1) $$I\propto P^{k}$$

with the k‐value being the exponent of the power dependence [49]. The k‐value provides qualitative insight into the underlying recombination mechanism. Sublinear power dependence (0<k<1) is commonly associated with defect‐mediated or trap‐limited recombination and may also reflect saturation effects. A nearly linear dependence (k≈1) is often observed for free neutral excitons or interlayer excitons under unsaturated conditions, whereas superlinear behavior (1<k<2) is commonly associated with multicarrier complexes such as trions or biexcitons [49]. These assignments should, however, be regarded as empirical guidelines rather than unambiguous signatures of a specific recombination pathway. To reduce the influence of saturation and laser‐induced heating at high excitation powers, we restricted the fitting between 0.1 and 4 mW in Figure 2c,f.

Our findings demonstrate effective exponents of k≈0.30 ($R^{2}=0.69$) for the AE and k≈0.35 ($R^{2}=0.97$) for the bare ${\mathrm{Cu}}_{2}O$ DE. Both values are strongly sublinear, consistent with trap‐mediated recombination. The similar k‐values indicate that the power‐law behavior alone does not conclusively distinguish the two emissions. However, the ${\mathrm{WS}}_{2}$/${\mathrm{Cu}}_{2}O$ emission saturates more rapidly, and its power dependence is therefore not fully captured by the power‐law model. This behavior is consistent with modified trap occupation at the heterointerface. Thus, the power‐dependent PL supports a defect‐mediated origin of the AE, while the assignment to a ${\mathrm{WS}}_{2}$‐modified interfacial pathway relies on the combined spatial, excitation‐dependent, temperature‐dependent, and bias‐dependent evidence. Moreover, the high width of the AE could be explained by these electrons relaxing radiatively to the aforementioned $\Gamma_{7^{+}}$ and $\Gamma_{8^{+}}$ valence bands that arise due to spin–orbit coupling, forming defect emissions ($I_{D1}$ and $I_{D2}$) depicted in Figure 2d. In bare ${\mathrm{Cu}}_{2}O$, excited charge carriers could be trapped in bulk defect states, and defect emissions are not confined to the surface states, leading to a less pronounced saturation behavior. It is also noteworthy that DE stemming from bulk ${\mathrm{Cu}}_{2}O$ could contribute to the observed AE, which might change the estimated k‐value. Future experimental and theoretical investigations are needed to discern this further and investigate the AE peak's components.

Furthermore, while local‐field effects can influence absolute PL intensities [50], they cannot explain the observed emission dynamics. The requirement for 457 nm excitation, consistent with high‐energy carrier injection, along with the rapid saturation under increasing optical power, indicates the dynamic filling and depletion of a finite interfacial state population rather than static optical interference.

To conclude, the low temperature and power‐dependent PL results suggest the interface‐modified defect emission (IMDE) facilitated by the ${\mathrm{WS}}_{2}$ monolayer is the cause of the observed anomalous emission in ${\mathrm{WS}}_{2}$/${\mathrm{Cu}}_{2}O$. In the following, we further investigate this conclusion by attempting to modulate this AE in a p‐n junction device made out of ${\mathrm{WS}}_{2}$/${\mathrm{Cu}}_{2}O$.

### 2.4 Modulating the Interface‐Modified Defect Emission

Using a device based on the ${\mathrm{WS}}_{2}$/${\mathrm{Cu}}_{2}O$ heterostructure (depicted in Figure 3a), we modulate the IMDE via a constant vertical voltage while PL measurements are simultaneously performed. The IMDE should be moderately tunable due to the persistent nature of defect trapping. If ILX formation were indeed present, strong IMDE quenching would be expected when bias is applied due to the ILX being torn apart by the applied bias [51].

**FIGURE 3:**
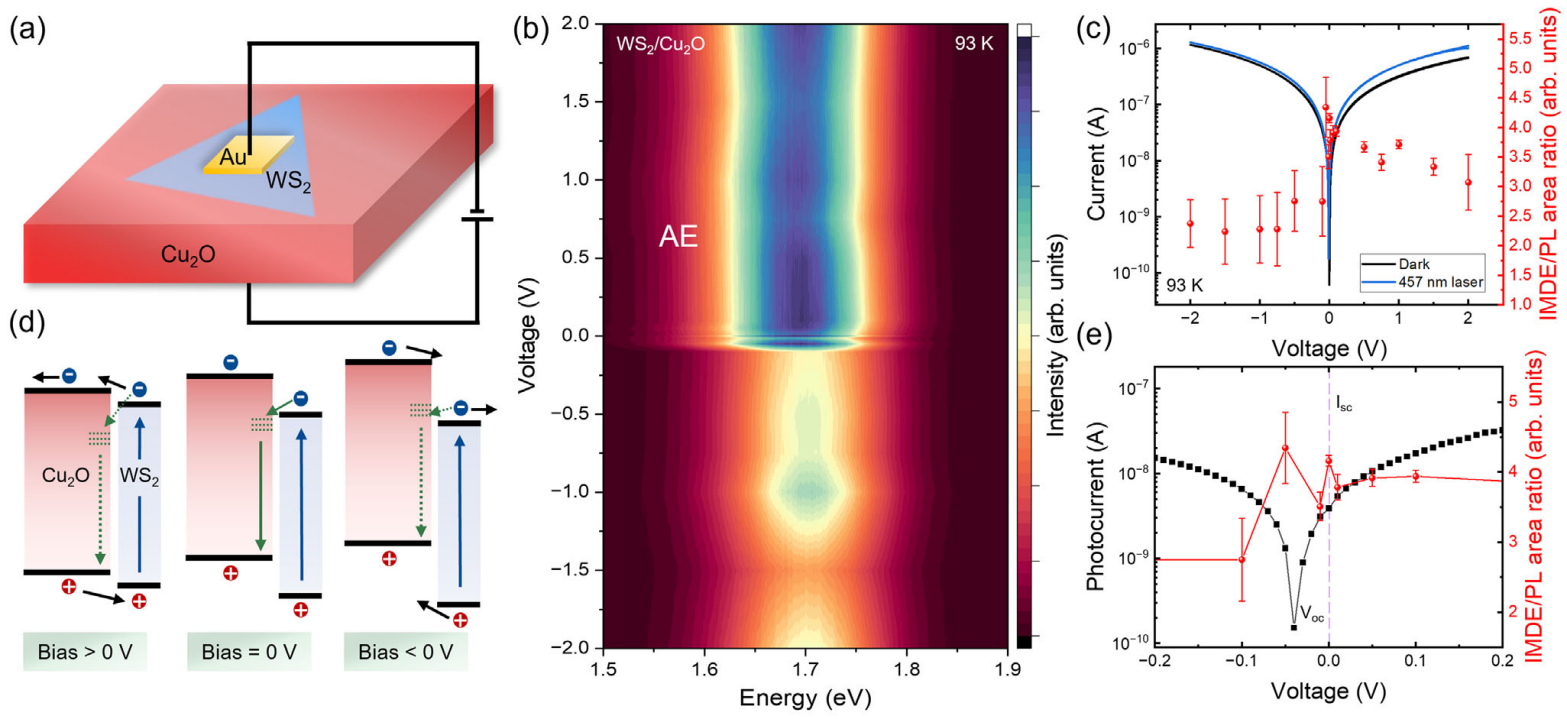
(a) A schematic of the contacted ${\mathrm{WS}}_{2}$/${\mathrm{Cu}}_{2}O$ heterostructure. (b) PL measurements are performed under applied voltage across the heterostructure at 93 K. (c) Current–voltage characteristics of the ${\mathrm{WS}}_{2}$/${\mathrm{Cu}}_{2}O$ heterostructure measured in the dark (black) and under 457 nm illumination (blue), overlaid with the normalized emission ratio R (red dots). The ratio exhibits an approximately 45% maximum‐to‐minimum modulation over the applied bias range. (d) A proposed model to describe the funneling of electrons from ${\mathrm{WS}}_{2}$ into ${\mathrm{Cu}}_{2}O$ surface defects. Under 0 V bias, this process is efficient. Applying a positive voltage forces electrons into ${\mathrm{Cu}}_{2}O$, momentarily enhancing this effect before they are driven into the ${\mathrm{Cu}}_{2}O$ bulk at higher voltages. Applying a negative bias forces electrons away from ${\mathrm{Cu}}_{2}O$, thereby rapidly reducing the photocurrent and quenching the AE effect. (e) The normalized IMDE/${\mathrm{Cu}}_{2}O$ PL area ratio (red) and the bias‐dependent electrical photoresponse (black), calculated as $I_{\mathrm{photo}}=\left|I_{\mathrm{light}}-I_{\mathrm{dark}}\right|$.

The results of this measurement at 93 K are shown in Figure 3b,c. The normalized IMDE/${\mathrm{Cu}}_{2}O$ PL area ratio exhibits an approximately 45% maximum‐to‐minimum modulation over the applied bias range and reaches a maximum near −0.05 V. In Figure 3c, we overlay the ratio of integrated IMDE and ${\mathrm{Cu}}_{2}O$ PL emissions (for normalization) over the current–voltage (I–V) characteristics of one representative device in the dark (black) and under 457 nm laser illumination (blue). The IMDE/PL area ratio exhibits asymmetric behavior around the −0.05 V peak, with the ratio decreasing more rapidly under negative applied bias than under positive applied bias. This suggests distinct mechanisms operating under different applied biases. Simultaneously, the I‐V curve measured under illumination shows a higher photocurrent contribution at positive bias compared to the negative bias, exactly where the IMDE/PL ratio decreases. This suggests a correlation between the IMDE and the generation of photocurrent in the ${\mathrm{WS}}_{2}$/${\mathrm{Cu}}_{2}O$ heterostructure. The IMDE modulation and current–voltage characteristics exhibited qualitatively consistent trends in both independent devices. Quantitative differences between the devices may arise from spatial variations in the ${\mathrm{Cu}}_{2}O$ interfacial defect density and in the defect landscape of CVD‐grown ${\mathrm{WS}}_{2}$. In particular, defects in ${\mathrm{WS}}_{2}$ may introduce competing non‐radiative recombination pathways before photoexcited carriers reach the interface [13, 48, 52]. Because the spectra were averaged before calculating the normalized emission ratio, the reported 45% modulation represents the response derived from the combined dataset rather than an average of independently calculated device modulation values. This investigation explores the IMDE and its electrical modulation under specific conditions and should be regarded as a proof‐of‐principle demonstration. Thus, an extensive statistical analysis of device‐to‐device variation is not included.

Conversely, bias‐dependent PL measurements of the ${\mathrm{WS}}_{2}$/${\mathrm{Cu}}_{2}O$ IMDE at room temperature show a red shift when voltage is applied from +2 V to −2 V compared to the ${\mathrm{Cu}}_{2}O$ DE (Figure S5a,c) and the ${\mathrm{WS}}_{2}$/${\mathrm{Cu}}_{2}O$ IMDE at 93 K (Figure S5e). Simultaneously, the relative intensity ratio does not show a significant modulation at room temperature (Figure S5b). The observed shift may arise from thermally activated lattice interactions that modify the occupation and spectral distribution of the defect‐related states [53]. Thermal phonon scattering may broaden the defect levels and make defect trapping and recombination less sensitive to small electric fields. Additionally, the thermal energy ($k_{B}T$) at room temperature may enable back‐transfer of charge carriers [44], further reducing the effect of the applied bias. The reduced intensity tunability at room temperature points to thermally activated dynamics. A higher density of suitable ${\mathrm{Cu}}_{2}O$ interfacial defect states may increase their bias‐dependent occupation through the ${\mathrm{WS}}_{2}$ sensitizing layer. This could improve room‐temperature control in future devices based on this heterostructure.

Using these results, we interpret this behavior with the model of ${\mathrm{WS}}_{2}$‐assisted interface‐modified defect emission as illustrated in Figure 3d. In the case of no bias (0 V), the transition of electrons from ${\mathrm{WS}}_{2}$ to ${\mathrm{Cu}}_{2}O$ and their subsequent radiative relaxation could happen efficiently at 93 K. In the case of small negative voltages (up to −0.5 V), the electrons are first accumulated in the ${\mathrm{WS}}_{2}$, and then predominantly captured by interfacial traps in the ${\mathrm{Cu}}_{2}O$. At larger negative voltages, the band bending rapidly extracts photoexcited electrons out of the ${\mathrm{WS}}_{2}$/${\mathrm{Cu}}_{2}O$ device, thus lowering the IMDE. When a positive bias is applied, faster carrier transport through the ${\mathrm{Cu}}_{2}O$ bulk reduces the probability of interfacial capture, leading to a decrease in IMDE intensity. Instead of decaying, the photoexcited electrons seem to contribute to the photocurrent in the device.

Finally, we address the low‐bias fluctuation in IMDE/${\mathrm{Cu}}_{2}O$ PL area ratio near 0 V observed in Figure 3e. The IMDE area ratio correlates with the photocurrent ($I_{\mathrm{photo}}$), which serves as a metric for the photoexcited charge carriers. $I_{\mathrm{photo}}$ is defined as:

(2) $$I_{\mathrm{photo}}=\left|I_{\mathrm{light}}-I_{\mathrm{dark}}\right|$$

where $I_{\mathrm{light}}$ is the current under illumination, and $I_{\mathrm{dark}}$ is the dark current. Two regimes are critical here: at the short‐circuit condition ($I_{\mathrm{sc}}$ at 0 V), the device operates in self‐powered mode. While the built‐in potential maximizes carrier extraction, the high flux of photogenerated carriers passing through the interface maintains a steady population of the interfacial defect states, resulting in a secondary local maximum in IMDE intensity.

At the open‐circuit voltage ($V_{\mathrm{oc}}$) point around −0.04 V, the bias compensates the internal field sufficiently to halt net carrier extraction across the ${\mathrm{WS}}_{2}$/${\mathrm{Cu}}_{2}O$ interface. As a result, photogenerated carriers likely accumulate in the ${\mathrm{WS}}_{2}$ rather than being swept away. This accumulation at the interface is expected to increase the occupation of the interfacial defect states responsible for IMDE generation, resulting in a maximum normalized IMDE/${\mathrm{Cu}}_{2}O$ PL average area ratio of 4.3±0.5 at −0.05 V. A further change in voltage to −0.1 V reduces the normalized average area ratio to 2.8±0.6. From the contact potential difference (CPD) in Figure S3a measured via KPFM, we use the estimated work functions (Figure S3b) of ${\mathrm{Cu}}_{2}O$ (4.01 eV) and ${\mathrm{WS}}_{2}$/${\mathrm{Cu}}_{2}O$ (4.15 eV) to calculate the maximum $V_{\mathrm{bi}}$ to be around −0.14 V, which correlates with the observed intensity reduction. The discrepancy between the estimated $V_{\mathrm{bi}}$ and the $V_{\mathrm{oc}}$ (−0.04 V) values may reflect Fermi level pinning by interfacial states in the buried ${\mathrm{WS}}_{2}$/${\mathrm{Cu}}_{2}O$ heterojunction [54], potentially pinning the Fermi level of ${\mathrm{WS}}_{2}$ and altering the charge transfer dynamics.

## 3 Conclusion

We characterized the electronic and optical properties of ${\mathrm{WS}}_{2}$/${\mathrm{Cu}}_{2}O$ heterostructures, focusing on a room‐temperature sub‐bandgap anomalous emission (AE) at 1.66 eV. Through a combination of temperature‐dependent and power‐resolved photoluminescence, we conclude that this emission likely originates from ${\mathrm{WS}}_{2}$‐assisted interface‐modified defect emission (IMDE) at oxygen‐related ${\mathrm{Cu}}_{2}O$ defect sites rather than a free interlayer exciton. The high carrier injection efficiency from the ${\mathrm{WS}}_{2}$ monolayer appears to enhance the population of these ${\mathrm{Cu}}_{2}O$ defect states.

Our device‐level study demonstrates an approximately 45% maximum‐to‐minimum modulation of the normalized IMDE/${\mathrm{Cu}}_{2}O$ PL area ratio over the applied bias range at 93 K, suggesting competition between radiative defect recombination and field‐driven carrier extraction. The related bias dependence of the normalized IMDE/${\mathrm{Cu}}_{2}O$ PL area ratio and the photocurrent near the flat‐band condition suggest the potential of this heterostructure for self‐powered optoelectronic applications. While our data show no strong evidence for interlayer exciton (ILX) formation competing with surface defect emission (DE), this possibility cannot be entirely ruled out and warrants further investigation, alongside future efforts to manipulate the Fermi level and control the IMDE using a gated contact. Future investigations utilizing thin‐film ${\mathrm{Cu}}_{2}O$/${\mathrm{WS}}_{2}$ architectures could not only suppress the bulk ${\mathrm{Cu}}_{2}O$ PL to highlight the defect‐dependent interfacial IMDE, but also enable the use of high‐resolution imaging techniques, such as STEM, to identify the specific local defect states governing these emission features.

More broadly, this work illustrates a complementary way of using mixed‐dimensional heterostructures. In many 2D/3D systems, the 2D semiconductor is the primary material of interest and the substrate is treated mainly as a perturbing environment. Here, the role is partly inverted: monolayer ${\mathrm{WS}}_{2}$ acts as an atomically thin sensitizer that enables optical and electrical access to weak ${\mathrm{Cu}}_{2}O$ surface and interfacial defect states. This perspective expands the function of 2D materials from active optoelectronic components to local probes of surface‐state physics in bulk semiconductors.

## 4 Experimental Section

### 4.1 Sample Fabrication

To grow ${\mathrm{WS}}_{2}$ using CVD, the tungsten precursor (aqueous solution composed of 3.2 mL of 100% ammonium metatungstate (AMT, *Sigma –Aldrich*), 2 mL of OptiPrep (*Sigma –Aldrich*), and 0.8 mL of DI water) was spin‐coated onto p‐doped Si substrates with a 285 nm ${\mathrm{SiO}}_{2}$. As a seeding promoter, we used Cholic acid sodium salt (*Sigma –Aldrich*), which was also applied via spin coating. We then annealed the substrates at 500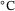 for 45 min in ambient conditions to convert AMT to tungsten trioxide ${\mathrm{WO}}_{3}$. The CVD growth process was performed in a three‐zone tube furnace (*ThermConcept*, ROK 70/750/12‐3z), schematically shown in Figure S2a. An argon (Ar) gas flow was established through a quartz tube at 500 sccm, passing from a gas source to an exhaust. Inside the tube, a combustion boat containing 350 mg of sulfur powder was placed in the first heating zone at 170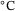. Downstream from the sulfur, the growth substrates were positioned on a custom alumina holder in the second zone. This zone was heated to 725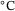 for 60 min to facilitate the reaction between tungsten trioxide (${\mathrm{WO}}_{3}$) and sulfur, forming tungsten disulfide (${\mathrm{WS}}_{2}$). A third zone at 600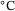 was used to help remove any reaction by‐products.

Single‐crystalline ${\mathrm{Cu}}_{2}O$ (111) substrates with the dimensions (1 mm × 5 mm × 5 mm) were commercially acquired from *SurfaceNet GmbH*. According to the manufacturer, the crystals were naturally sourced and were epi‐polished to achieve the (111) crystal facet.

4.1.1

The CVD‐grown ${\mathrm{WS}}_{2}$ flakes were transferred onto the ${\mathrm{Cu}}_{2}O$ substrates using a polystyrene‐assisted process, based on the work by Gurarslan et al. [55]. A toluene‐polystyrene solution (9 g/100 mL) was spin‐coated onto the growth substrate. After annealing at 80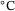, a polystyrene layer was formed, which was then detached using deionized (DI) water. The detached layer, with the ${\mathrm{WS}}_{2}$ flakes on it, was carefully transferred onto the target substrate (${\mathrm{Cu}}_{2}O$). To ensure good contact and minimize strain, the samples were annealed in two steps: first at 85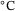 for 90 min to remove any water residue, and then at 150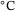 for 30 min to relieve transfer‐induced strain. Finally, the polystyrene was dissolved by immersing the samples in toluene for 25 min, followed by a wash with isopropyl alcohol (IPA).

### 4.2 Optical and Electrical Measurements

We used a *WITec alpha300 RA* confocal Raman spectrometer for our steady‐state photoluminescence (PL) and Raman measurements. Depending on the measurement, we used a λ=532 nm or λ=457 nm laser with a power output of 1 mW unless otherwise specified, with an N.A. of 0.75. For PL measurements, we used a 300 ${\mathrm{lmm}}^{-1}$ grid size, while the Raman measurements were performed using a 1800 ${\mathrm{lmm}}^{-1}$ grid. A *Linkam THMS350EV* heating and cooling stage was controlled by an external temperature system and was integrated with the spectrometer for temperature‐dependent measurements. Overall, four ${\mathrm{Cu}}_{2}O$ samples, each containing at least two flakes, were used to perform PL and Raman measurements.

Electrical measurements were performed on two independent ${\mathrm{WS}}_{2}$/${\mathrm{Cu}}_{2}O$ devices, using the *Linkam THMS350EV* stage coupled with a *Keithley 2612B* source‐measure‐unit under vacuum conditions ($1\times 10^{-2}$ mbar). The electrical contact (10 nm Cr, 100 nm Au) on top of the ${\mathrm{WS}}_{2}$ was fabricated using a standard photolithography process, using a maskless optical lithography machine *SMART PRINT UV* from *microlight3D*. Cr and Au were deposited using electron‐beam and thermal evaporation at $1\times 10^{-6}$ mbar, respectively. For the bias‐dependent analysis, four spectral measurements from two independent devices were averaged at each applied voltage before integration. The normalized emission response was then defined as $R=A_{\mathrm{IMDE}}/A_{{\mathrm{Cu}}_{2}O}$, where $A_{\mathrm{IMDE}}$ and $A_{{\mathrm{Cu}}_{2}O}$ are the integrated areas of the IMDE and ${\mathrm{Cu}}_{2}O$ PL bands, respectively, in the averaged spectrum. The maximum‐to‐minimum relative modulation over the applied bias range from −2 to +2 V was calculated as $\left(R_{\max}-R_{\min}\right)/R_{\max}\times 100\%$, yielding approximately 45%. The data processing, including the statistical analysis, was performed using *OriginPro*.

Time‐resolved photoluminescence (TR‐PL) measurements were performed using a time‐correlated single photon counting (TCSPC) setup. Excitation was done by a pulsed diode laser (*PicoQuant LDH‐P‐C‐405*, λ=405 nm) driven by a *PDL 800‐D* driver at a repetition rate of 20 MHz. The emission was detected by a single‐photon avalanche diode detector connected to the TCSPC module. *OriginPro* was used to fit the data, using $I\left(t\right)=y_{0}+A_{1}e^{-\frac{t-t_{0}}{t_{1}}}+A_{2}e^{-\frac{t-t_{0}}{t_{2}}}$.

### 4.3 X‐Ray Photoelectron Spectroscopy

To perform the XPS measurements included in this work, *VersaProbe II (Ulvac‐Phi)* XPS system with Al Kα radiation with an excitation energy of 1486.74 eV was used. Data analysis was conducted using *CasaXPS* software. The data were fitted using the LA(50) (Lorentzian‐Asymmetric) line shape and Shirley background subtraction.

### 4.4 Kelvin Probe Force Microscopy

Kelvin Probe Force Microscopy (KPFM) was performed under ambient conditions using an integrated setup within the *WITec alpha300 RA* confocal Raman spectrometer. The measurements utilized a two‐pass technique: in the first pass, the topography was recorded using tapping mode. In the second pass, an AC voltage with a frequency of 100 Hz was applied between the sample and the tip, inducing cantilever oscillations via electrostatic forces. All data were analyzed using the Gwyddion software. The sample work function was determined using $\Phi_{s}=\left|q\right|\left(V_{\mathrm{CPD},\mathrm{Au}}-V_{\mathrm{CPD},s}\right)+\Phi_{\mathrm{Au}}$, using gold contacts as a reference ($\Phi_{\mathrm{Au}}=4.7$ eV) [56].

## Conflicts of Interest

The authors declare no conflicts of interest.

## Supporting information


**Supporting File**: smll75662‐sup‐0001‐SuppMat.pdf.

## Data Availability

The data that support the findings of this study are openly available in a repository at https://doi.org/10.17172/nomad.wkqs‐gwmm.
