# Supplementary material for: Anomalous Sub‐Band Gap Emission at Room Temperature in Mixed‐Dimensional WS2/Cu2O Heterostructures
Source: Small. 2026 Sep 21;22(62):e75662. doi: 10.1002/smll.75662 (PMC13742907; doi:10.1002/smll.75662)
Supplement: Supplementary file 1 [file SMLL-22-e75662-s001.pdf]

Supporting Information to "Anomalous Sub-band Gap Emission at Room Temperature in Mixed-Dimensional WS_2/Cu_2O Heterostructures"

Osamah Kharsah, Jonah von Kuczkowski, Ahmed Shahin, Rouaa Albalkhi, Aya Al-Amwi, Ibrahim Titi, Yossarian Liebsch, Oliver Altenhoff, Ulrich Hagemann, Anke Hierzenberger, Kevin Musselman, and Marika Schleberger

Date: August 10, 2026.

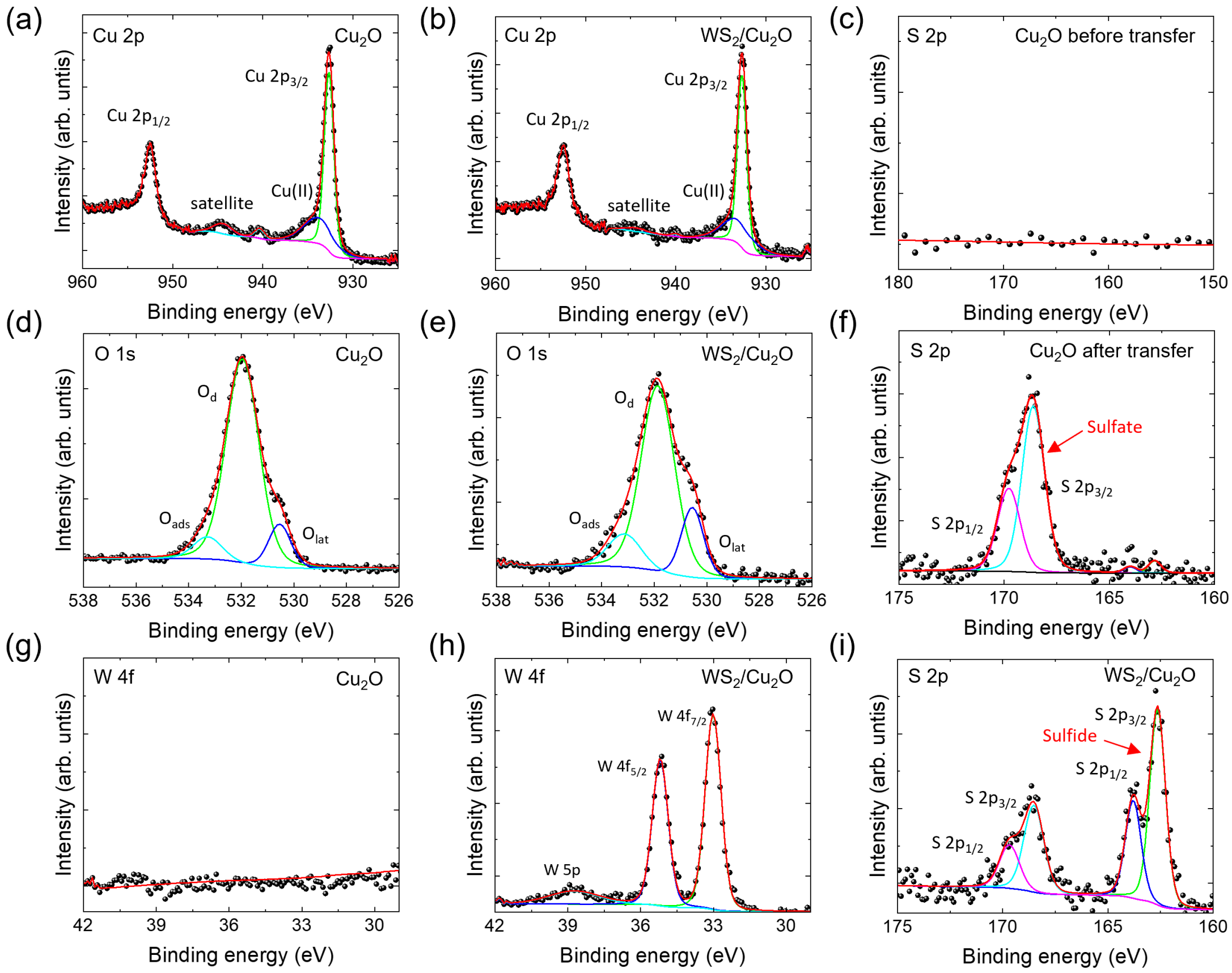

FIGURE S1. **(a)** The Cu 2p core level of Cu_2O, showing the Cu $2p_{1/2}$ and Cu $2p_{3/2}$ peaks, as well as the characteristic satellite features. **(b)** The Cu 2p spectrum remains unaltered in the WS_2/Cu_2O heterostructure, exhibiting a slightly lower signal-to-noise ratio. **(c)** Control S 2p core level for bare Cu_2O before the transfer of WS_2. No S $2p_{3/2}$ and S $2p_{1/2}$ peaks are observed. **(d)** The O 1s core level for Cu_2O reveals the oxygen lattice state (O_{lat}), the oxygen-related defect state (O_d) (or corresponding adsorbed OH-groups), and adsorbed water species (O_{ads}). **(e)** In WS_2/Cu_2O, the O_d component shows a reduced area ratio compared to bare Cu_2O. **(f)** The S $2p_{3/2}$ and S $2p_{1/2}$ peaks observed in bare Cu_2O after the WS_2 transfer indicate the presence of sulfur, attributed to sulfate species (SO_4^{2-}) formed during the WS_2 growth. **(g)** Control W 4f core level for bare Cu_2O (showing no signal) and **(h)** the WS_2 monolayer. **(i)** In the WS_2/Cu_2O heterostructure, the S 2p peaks from the WS_2 monolayer are clearly distinguishable from the sulfate species present on the WS_2-free regions of the Cu_2O substrate after the transfer.

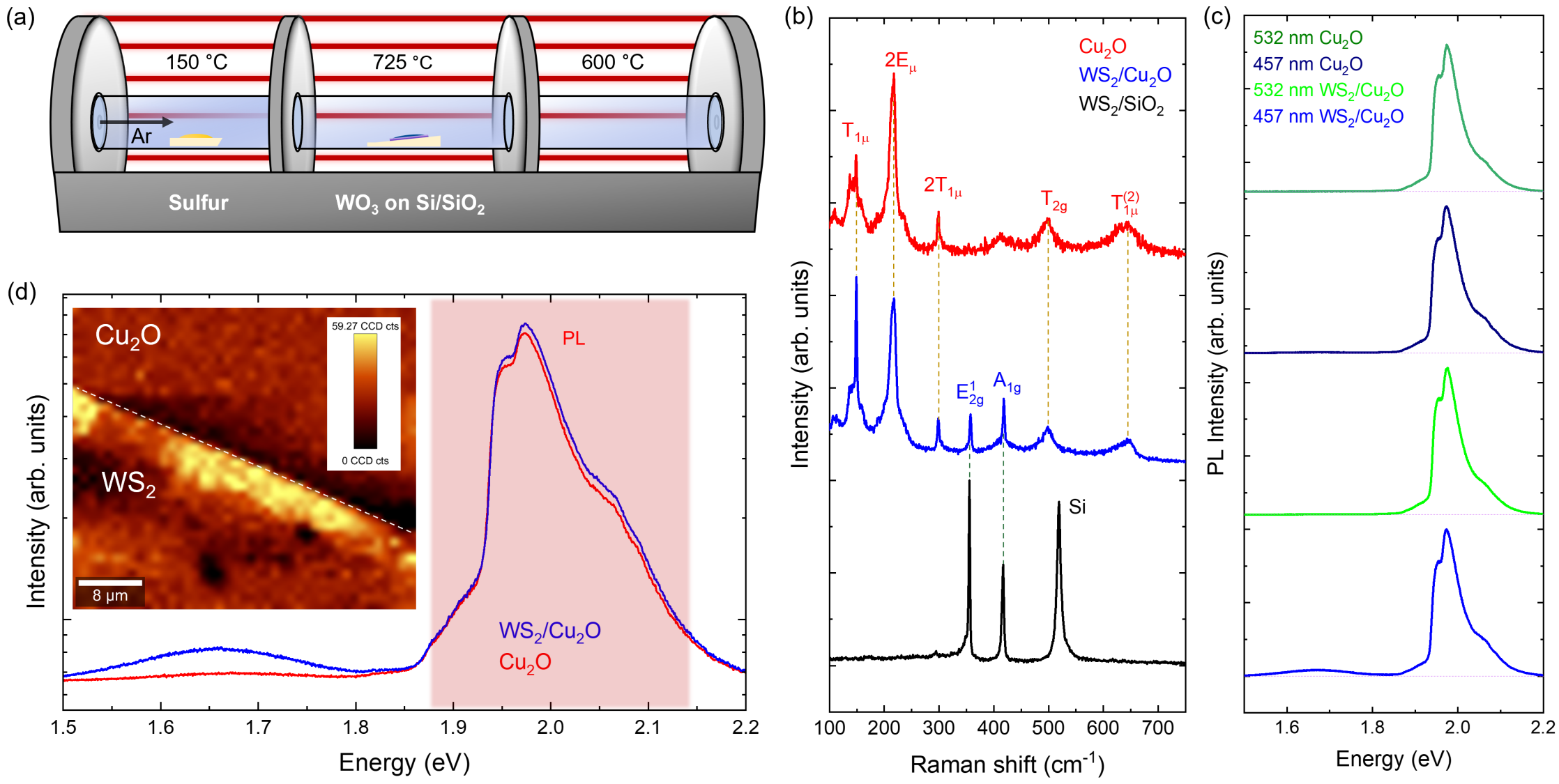

FIGURE S2. **(a)** Illustration of the CVD process used to grow WS_2 monolayers. **(b)** Raman spectra of WS_2, Cu_2O, and the WS_2/Cu_2O heterostructure acquired with 457 nm excitation. **(c)** Comparison of PL spectra acquired with 532 nm and 457 nm excitation from bare Cu_2O and WS_2/Cu_2O regions. **(d)** Spatially resolved PL map of the Cu_2O band gap emission, showing that the dominant Cu_2O PL originates from the substrate and is spatially distinct from the 1.66 eV sub-band-gap emission associated with the WS_2-covered region.

(a)

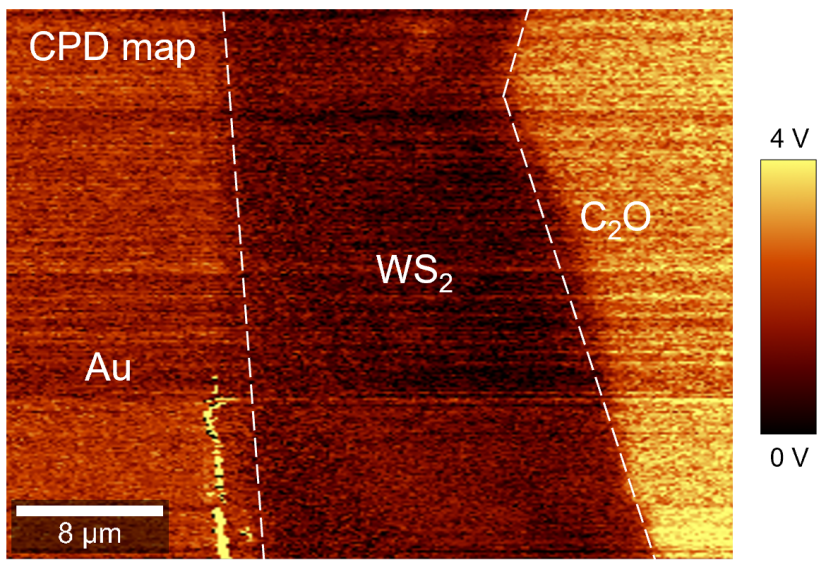

(b)

KPFM	Au	WS_2/Cu_2O	Cu_2O
Average CPD	0.61 V	0.55 V	0.69 V
Work function	4.7 eV	4.15 eV	4.01 eV

(c)

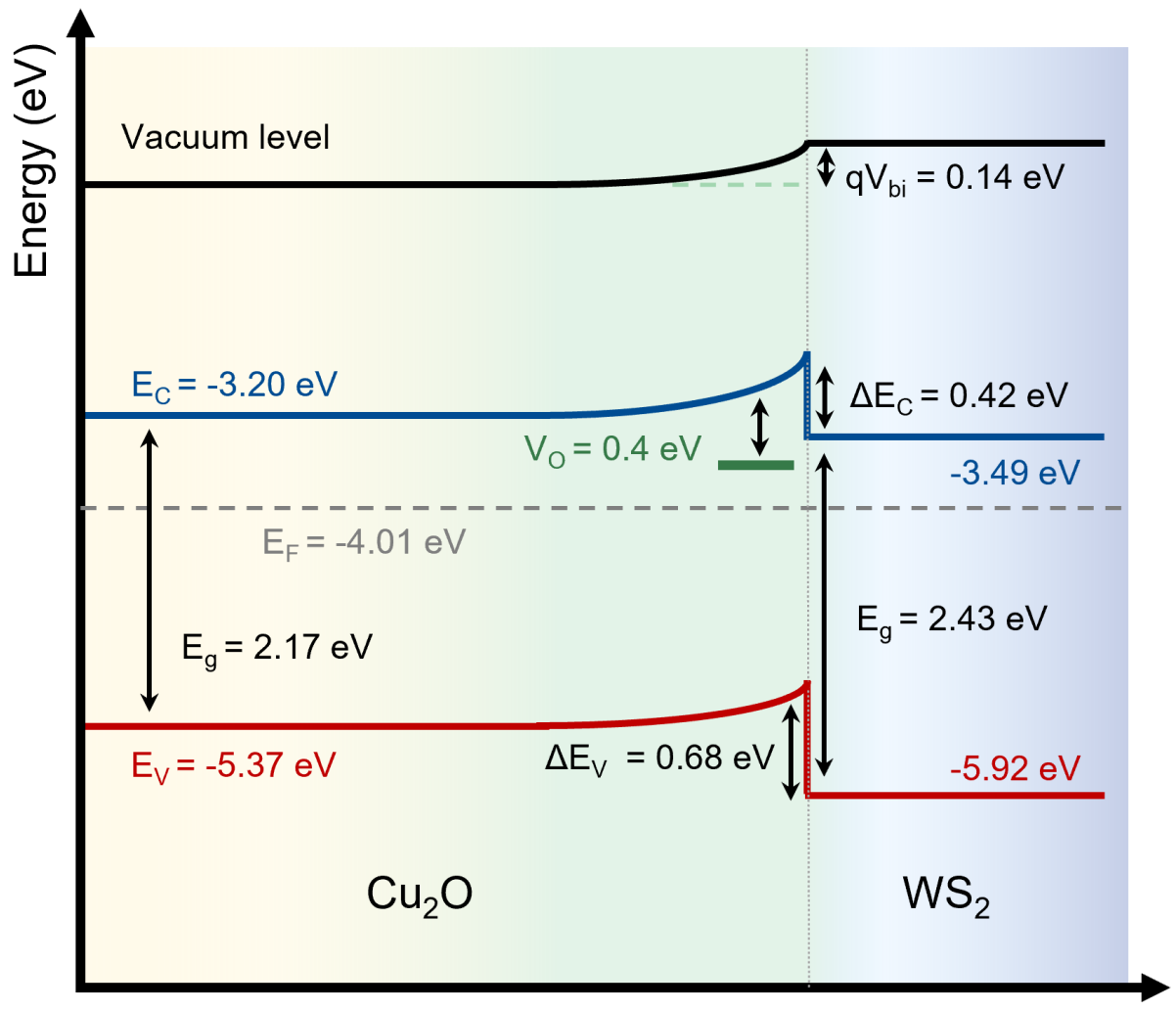

FIGURE S3. **(a)** A CPD map of WS_2/Cu_2O, bare Cu_2O, and a gold contact used as a reference. The tabular form in **(b)** contains the average CPD values for the three materials, as well as the calculated work function. The equation used for the calculation of the work function is $\Phi_s = |q|(V_{CPD,Au} - V_{CPD,s}) + \Phi_{Au}$, using Au contacts as a reference ($\Phi_{Au} = 4.7$ eV). **(c)** Estimated type-II band alignment of the WS_2/Cu_2O heterojunction, derived from literature values for electron affinities and band gaps combined with the measured KPFM work functions. This alignment is used only as a qualitative estimate because ambient KPFM can be influenced by adsorbates, transfer residues, and local interface dipoles.

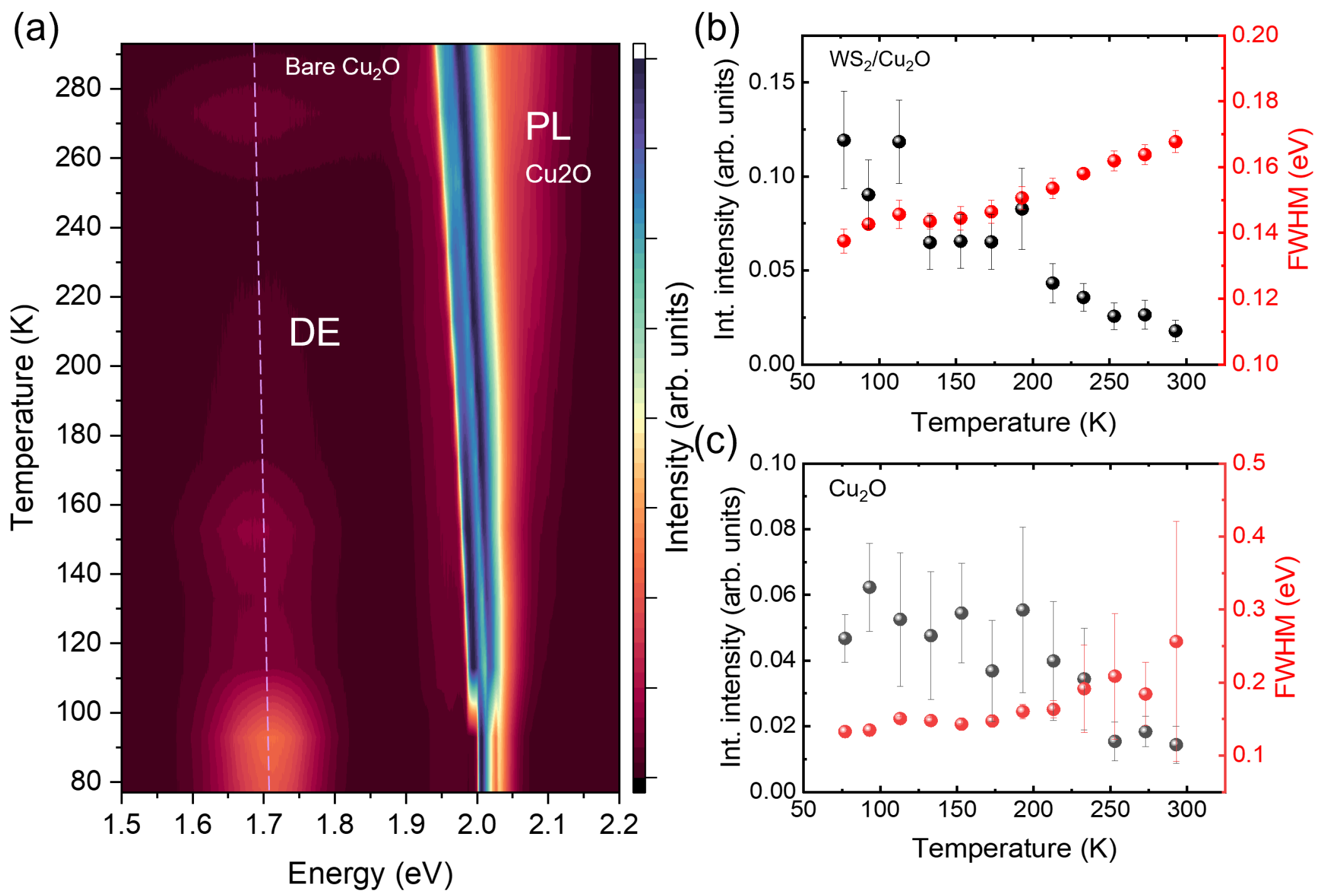

FIGURE S4. **(a)** Temperature-dependent PL map of bare Cu_2O. **(b)** Integrated PL intensity and FWHM of the AE in WS_2/Cu_2O as a function of temperature. **(c)** Integrated PL intensity and FWHM of the DE in bare Cu_2O as a function of temperature. The standard error in the statistical analysis is based on at least six spectra per temperature acquired from at least two WS_2 flakes or bare Cu_2O sites distributed across four independent Cu_2O substrates.

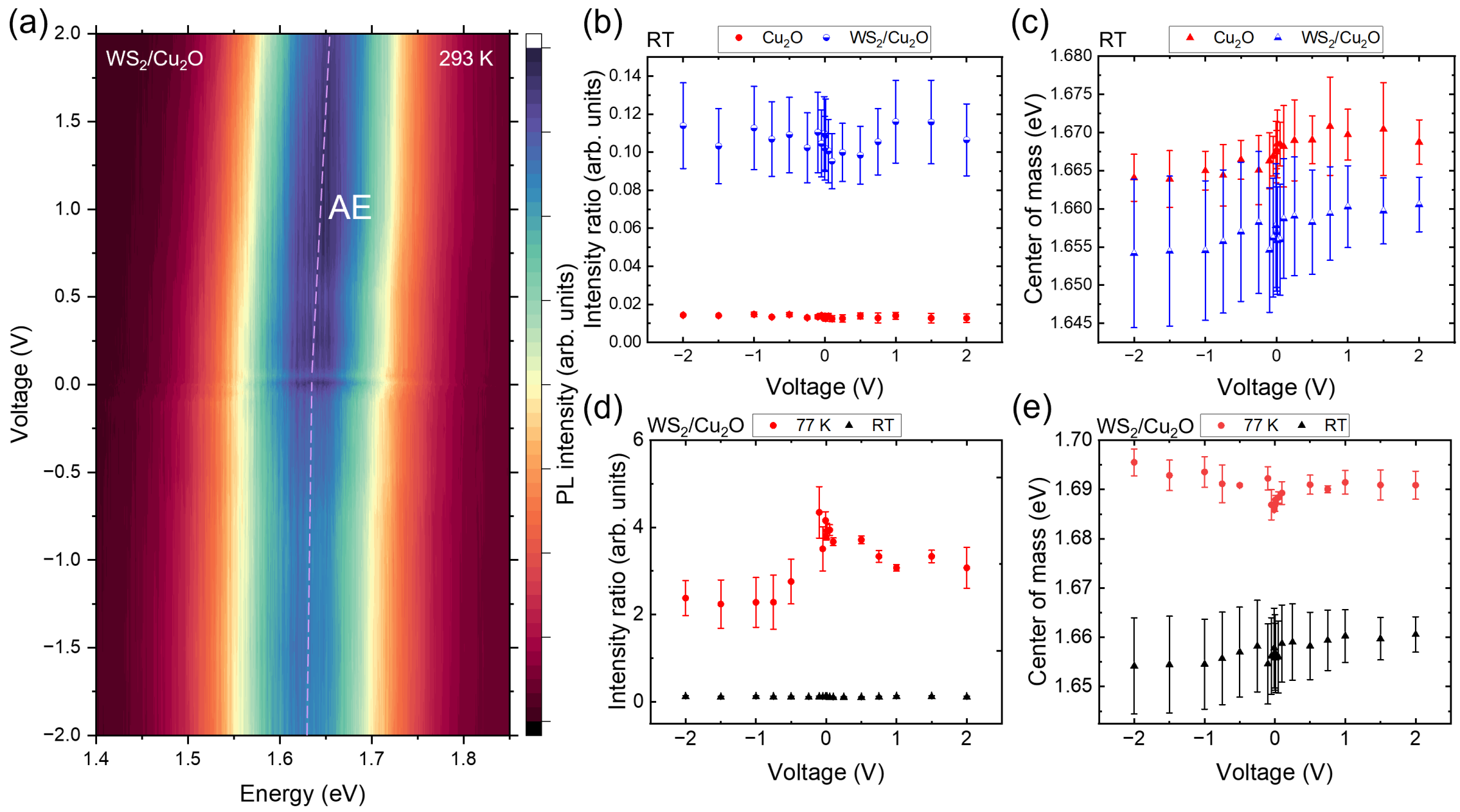

FIGURE S5. **(a)** Bias-dependent PL spectra of the WS_2/Cu_2O heterostructure at room temperature. **(b)** Integrated emission intensity ratios of WS_2/Cu_2O (blue half-filled symbols) and bare Cu_2O (red solid symbols) at room temperature. **(c)** Emission center of mass of WS_2/Cu_2O (blue half-filled symbols) and bare Cu_2O (red solid symbols) at room temperature. **(d)** Comparison of the integrated WS_2/Cu_2O emission intensity at room temperature (black triangles) and 93 K (red circles). The reduced tunability at room temperature is attributed to thermally activated carrier redistribution and reduced field sensitivity of defect occupation. **(e)** Emission center of mass of WS_2/Cu_2O at room temperature (black triangles) and 93 K (red circles). The standard error in the statistical analysis is based on four measurements taken from two independent devices.
